# Development of Computed Echo Tomography—An Imaging Breakthrough Addressing the Limitations of Conventional Ultrasound: A Baseline Imaging Analysis for Traumatic Injuries

**DOI:** 10.1016/j.acepjo.2025.100181

**Published:** 2025-05-23

**Authors:** John Cheronis, Michael Cronan, Dare Nwaka, Matthew Bradley, Paul K. Carlton, Rosemary Kozar, John McGahan, Melissa Myers, Elizabeth Powell, David Specht

**Affiliations:** 1 MAUI Imaging, Inc, Tucson, Arizona, USA; 2 Diagnostic Medical Sonography Program, University of Maryland Baltimore County, Baltimore, Maryland, USA; 3 Departments of Surgery and Emergency Medicine, Uniformed Services University of the Health Sciences, Bethesda, Maryland, USA; 4 Surgeon General of the Air Force (Retired), United States Armed Forces, College Station, Texas, USA; 5 Shock Trauma Center, University of Maryland School of Medicine, Baltimore, Maryland, USA; 6 Department of Radiology, University of California Davis Medical Center, Sacramento, California, USA

**Keywords:** point-of-care ultrasound, POCUS, trauma imaging, eFAST

## Abstract

**Objectives:**

The diagnosis and triage of trauma in austere environments using ultrasound can be severely limited by bone and other obstructions, particularly when dealing with intracranial, spinal, thoracic, and long bone injuries. A novel form of ultrasound, computed echo tomography (CET), may provide for more complete “whole body” imaging capability, thereby significantly improving patient management.

**Methods:**

To document and assess the imaging capabilities of the recently Food and Drug Administration-cleared CET system (MAUI Imaging K3900), we conducted 3 whole-body imaging sessions using 6 normal volunteers. Sixty-five predefined views of 4 different anatomic regions were obtained at each session. Images were scored by 5 clinicians experienced in trauma/general surgery, emergency medicine, and/or interventional radiology using the American College of Emergency Physicians diagnostic image quality scoring system. Imaging scores ≥3 were deemed adequate for inclusion in a “head-to-toe” imaging protocol being developed for the US military.

**Results:**

Overall, 59 views (90.8%) were deemed adequate for clinical decision making. Eleven (16.9%) had average scores between 3 and 4; and 48 (73.8%) had average scores ≥4. Imaging the cranial vault demonstrated numerous anatomic details. Extremity imaging revealed detailed views of both the boney cortex and the medullary cavity. Abdominal imaging showed clear views of the liver, spleen, and kidneys without any rib artifacts.

**Conclusion:**

CET-based imaging eliminates bone-related artifacts thereby allowing access to critical brain and extremity imaging and removes rib shadows from thoracic and abdominal organ imaging. CET imaging deserves further investigation for field-based trauma diagnosis and general imaging in other resource-limited environments.

**The Bottom Line**

Computed echo tomography (CET) is a new form of ultrasound that uses the same frequencies and energies as conventional ultrasound but differs in the way energy is introduced into the body and how the resulting echoes are captured and interpreted. CET can image through bone, gas, and other obstructions that confound conventional ultrasound, allowing imaging in areas of the body that previously have only been visualized with computed tomography and/or magnetic resonance imaging, such as the cranial vault and mediastinum. CET may provide for the development of a whole-body trauma evaluation protocol for diagnosing and managing traumatic injuries in resource-limited environments.

## 1 Introduction

### 1.1 Background

State-of-the-art evaluation of multitrauma victims in the United States requires immediate access to sophisticated imaging systems (computed tomography [CT], magnetic resonance imaging [MRI], x-ray, and ultrasound) and trained professionals capable of performing and interpreting these studies. This is a major problem facing health care providers in rural and other resource-limited settings without immediate access to level 1 or level 2 trauma facilities.^1^ In addition, patients with unstable trauma cannot be safely transported out of the Emergency Department, making it difficult to access advanced imaging modalities. Point-of-care ultrasound (POCUS) is invaluable in the evaluation of patients with trauma but is severely limited in the evaluation of intracranial and mediastinal injuries by its inability image through bone.

Unfortunately, all currently available POCUS systems have significant inherent limitations that prevent them from being used to assist in the evaluation of intracranial pathology and mediastinal injuries. The 2 most important limitations of all conventional ultrasound systems are the acoustic impedance mismatches (bone, gas/air, metal instruments, and foreign bodies), which cause significant problems with absorption, reflection, and scattering, resulting in the inability to image deeper structures and the significant ultrasound training and experience needed to find the right acoustic windows.

### 1.2 Importance

Computed echo tomography (CET) is a POCUS system that obviates many of these limitations. The unique configuration of the CET ultrasound transducer and the unfocused nature of the transmitted ultrasound beam allows for improved imaging around most traditional barriers. As a result, positioning of both the ultrasound transducer and the patient is crucial for optimal image capture. This poses a significant challenge in the patient with trauma, where optimal positioning may be difficult or impossible. Also, imaging anatomy that cannot be seen by conventional ultrasound (eg, intracranial) is possible. Moreover, the image capture process can be significantly easier to perform as searching for adequate windows is no longer necessary. Consequently, the amount of training required to become proficient in image acquisition with CET may be significantly less than for traditional ultrasound.

**Figure 1:**
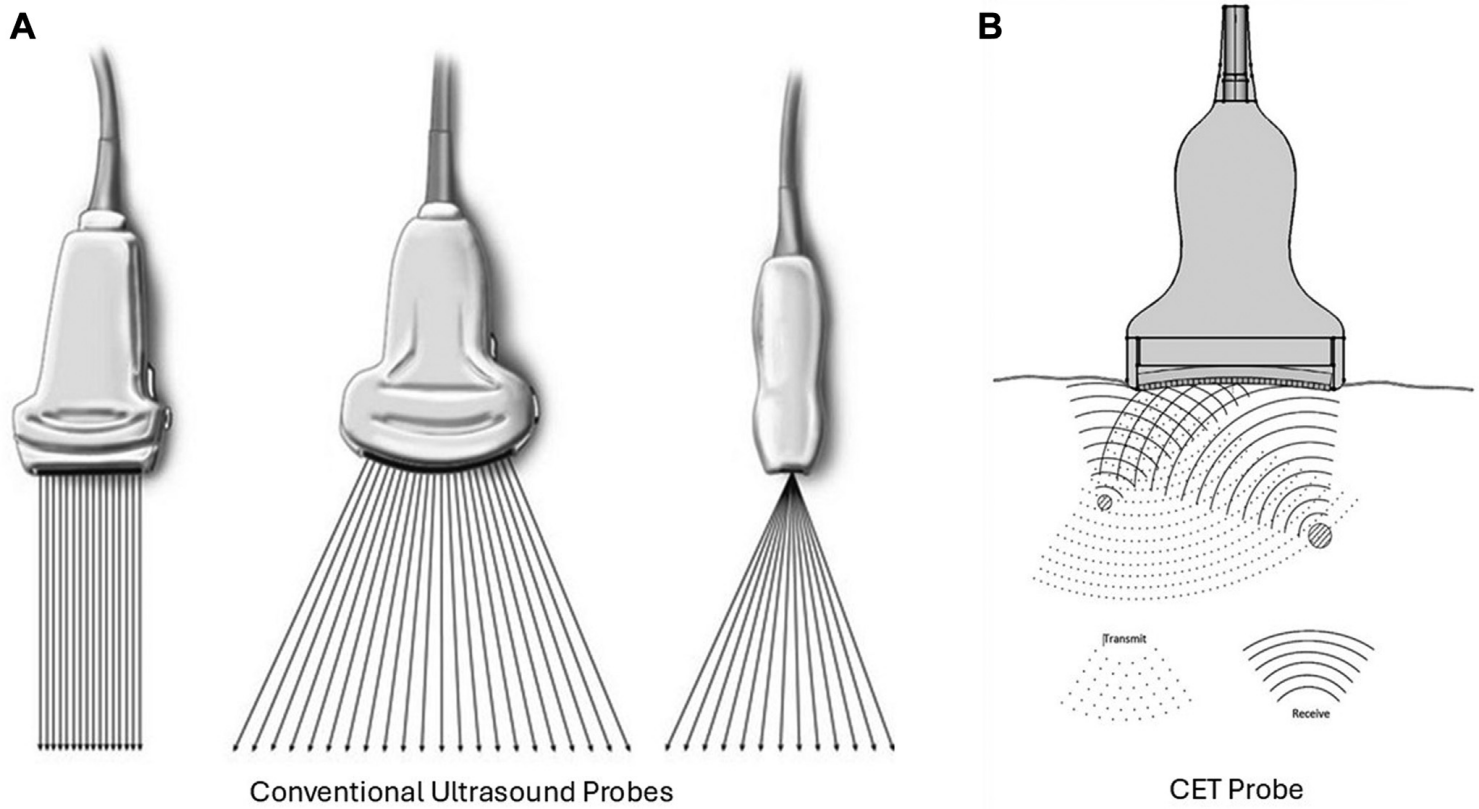
(A) Conventional/traditional ultrasound. Traditional ultrasound requires that key parameters (eg, depth, zoom area, and dynamic range) be predetermined before sending ultrasound energy out of the probe. The ballistic pulses based on these parameters are then sent out and predetermine how beamforming will be done to create images (ie, beamforming on the *send* side). (B) Computed echo tomography (CET) ping-based imaging. With CET, the key ultrasound parameters (eg, depth, zoom area, and dynamic range) do not need to be predetermined. Unlike conventional ultrasound systems that use beam line acquisition, CET systems transmit unfocused acoustic pings from element groups and receive the reflected echoes on all elements of the array. This method enables the entire field of view to be insonified and reconstructed with only a few pings, enabling high frame rates. A unique aspect of CET's receive side beamforming algorithm adjusts for the numerous flight paths to bring all the data into phase (ie, beamforming on the *receive* side). Each reconstructed pixel is fully focused in transmit and receive, significantly improving resolution and contrast compared with fixed transmit focusing of conventional systems. Finally, the ping-based method naturally enables adaptive beamforming techniques, which includes correcting for sound speed errors and blocked elements, further improving image quality, especially for difficult use cases such as transcranial imaging or imaging across ribs.

Conventional phased array technology is widely used in ultrasound imaging to produce a steerable and focused beam along a specified line of sight known as a “scan line” (Fig 1A). Scan lines are then aggregated together to form an image.^2^ This method of interpreting echoes from tissue along scan lines works very well when all of the tissues share a similar speed-of-sound (eg, all soft tissues). In contrast to conventional ultrasound, CET employs ping-based imaging (Fig 1B) whereby an unfocused transmit wave is sent via a single ping (or a combination of individual pings). With CET ping-based imaging there is no specialized transmit-side beamforming, which allows for dynamic focus at all depths. Echoes returning from the tissues are received by array elements with accommodations made for time-of-flight variation in the returning pathways. This accommodation then allows echoes to be aligned and compounded in real-time, overcoming speed-of-sound variations. Consequently, CET data sets enable images to be formed in regions that would normally be obscured, for example, behind a rib, the sternum, or in the cranial vault.

### 1.3 Goals of This Investigation

Based on the potential for imaging in anatomic regions that are currently *“terra incognita”* for traditional ultrasound we have embarked on a 3-phase program to assess CET for the development of a full “head-to-toe” trauma evaluation protocol (the CET focused assessment with sonography for trauma (FAST) examination protocol) that can be implemented anywhere that 110 to 220V power is available (the system’s sole requirement). The first phase, phase 0, was intended to establish the baseline imaging capabilities of the MAUI K3900 CET system to provide a benchmark for assessing the improvements made during the development program. This report is a summary of the results obtained with the system as of June 2024. The K3900 was cleared for clinical use and commercial sales by the US Food and Drug Administration (FDA) in September 2023.

## 2 Methods

### 2.1 Study Design

Baseline imaging assessment of 65 predefined views of normal anatomy in 4 anatomic regions (head/neck, chest, abdomen/pelvis, and the long bones of the upper and lower extremities) by a panel of 5 expert clinicians experienced in trauma/general surgery (M.B., P.K.C., and R.K.), emergency medicine (M.M.), and diagnostic ultrasound and/or interventional radiology (J.M.) was conducted over a 4-month period (March-June 2024).

### 2.2 Interventions/Exposures

Imaging was conducted by experienced sonographers (M.C. or D.N.) at 3 separate locations using 6 normal volunteers (2 at each site and a total of 4 male and 2 female subjects) recruited by word of mouth at each of the sites. All images were obtained using a 3 MHz concave probe. Imaging was conducted under a Western Institutional Review Board - Copernicus Group and Department of Defense Office of Human Research Oversight approved protocol.

### 2.3 Measurements

Image quality was assessed independently in real-time using the American College of Emergency Physicians diagnostic image quality scoring system.^3^ The reviewing clinicians were instructed to assess the images obtained for their ability to provide a view of the underlying anatomy that would be adequate to make a diagnosis of a traumatic injury based on their clinical experience.

### 2.4 Outcomes

Views with image quality scores ≥3 were deemed to be of adequate quality to advance into phase 1. Comparative imaging using conventional ultrasound was not included in this study. Unfortunately, not all views were able to be assessed by all 5 clinicians because of unexpected technical difficulties (a problem with the software controlling the cooling fan) experienced during 2 of the imaging sessions. However, all views were assessed by a minimum of 3 of the 5 clinicians. The technical issues experienced have been corrected and have not been experienced again.

## 3 Results

**Figure 2:**
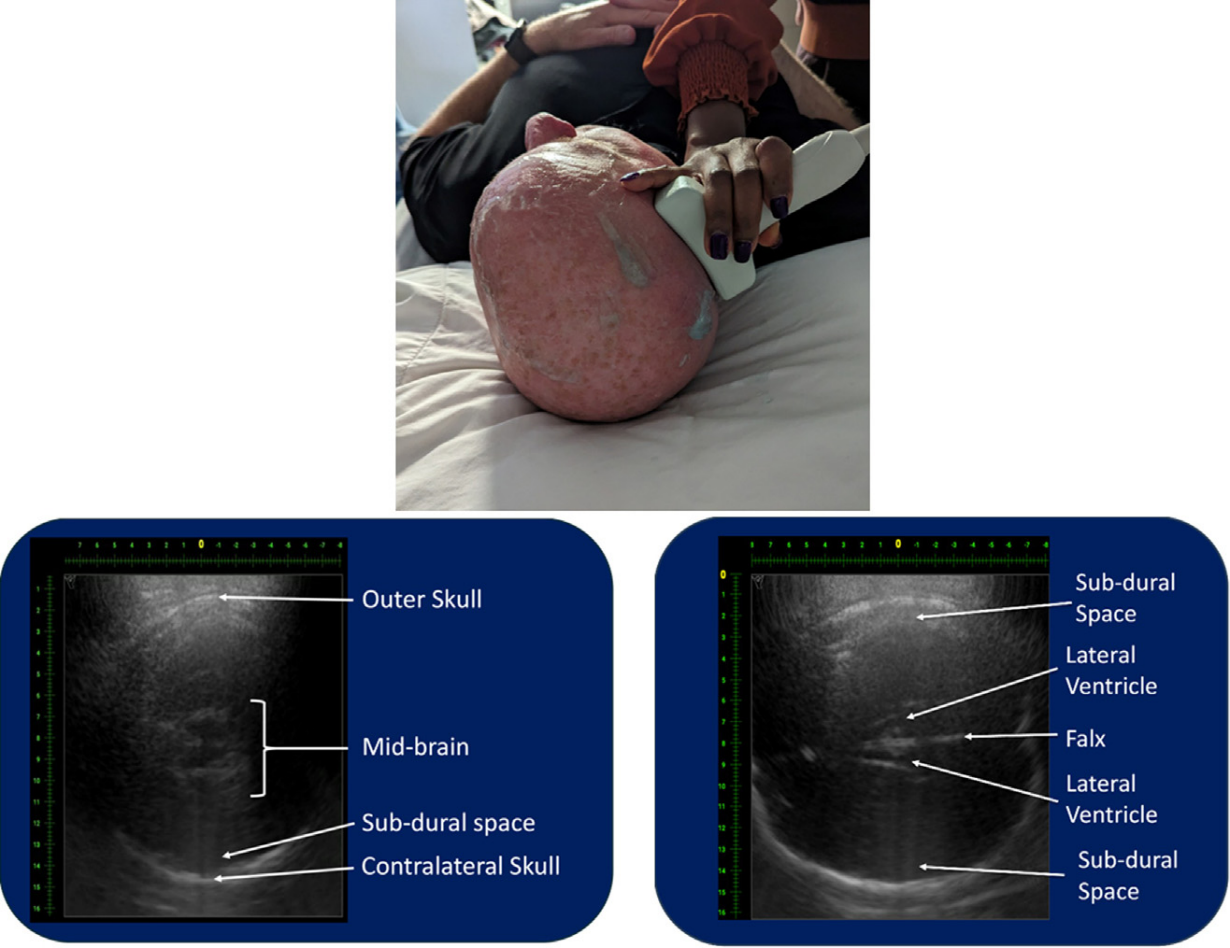
Transcranial image of an adult male cranium. A 3 Mhz probe was placed on the right squamosal bone above the ear. The image on the left was obtained by aiming the probe across the skull to the corresponding region on the contralateral side. Using this approach both the ipsilateral and contralateral skull and subdural regions as well as the midbrain can be easily visualized. The image on the right was obtained using the same probe position but aiming the probe upward until the falx and lateral ventricles come into view.

**Figure 3:**
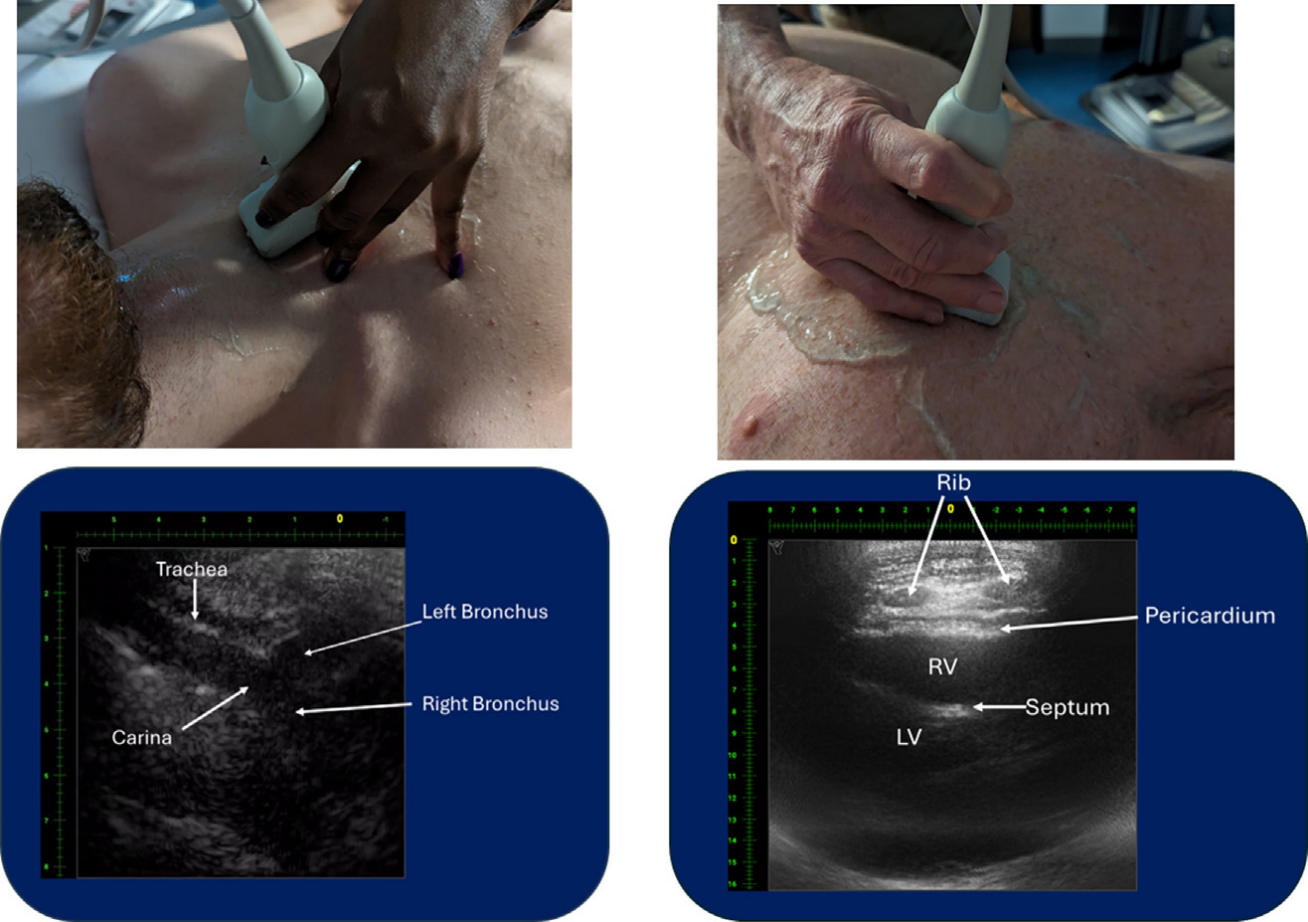
Trachea, bronchi, and heart imaged through the sternum and ribs. The image on the left was obtained by placing the probe in the middle of the upper chest with the probe extending from the suprasternal notch, over the manubrium, and over the upper portion of the body of the sternum. The image on the right was obtained by placing the probe over the left parasternal region and angled along the axis of the heart. This view of the pericardium and heart is significantly easier to obtain with the concave MAUI probe than the subxiphoid view and would be the preferred image for use in the eFAST examination. Note the oval shape of the ribs when viewed in cross-section with no rib shadowing.

The Table is a summary of the scores for the 65 views of the 4 different anatomic regions. Overall, only 6 views (9.2%) were deemed inadequate for clinical decision making. Eleven (16.9%) had average scores between 3 and 4; and 48 (73.8%) had average scores ≥4. Not surprisingly, 5 of the 6 views that were scored as being inadequate were in anatomic regions where bone is immediately below the skin (skull, sternum, and cervical spine). Improvements in imaging in these areas are ongoing. The other view considered to be inadequate was the subxiphoid view of the heart, which is part of the standard FAST^4^, ^5^, ^6^ or extended FAST (eFAST)^7^ examinations. This was primarily a result of difficulties in obtaining adequate probe-skin contact due to the concave design of the CET probe. Representative images of selected views that are not possible using conventional ultrasound (intracranial and mediastinal images) are provided in Figures 2 and 3.

**Table:** American College of Emergency Physicians grades for 65 views of 4 anatomic regions using computer echo tomography (CET).

View	Reviewer					AVG
	1	2	3	4	5	
eFAST						
Right Upper Quadrant	5	4	4	4	5	4.40
Left Upper Quadrant	4	4	4	4	5	4.20
Pelvis	5	5	4	5	2	4.20
Cardiac subxiphoid	NA	2	2	3	2	2.25
Cardiac left parasternal	5	5	4	4	5	4.60
Right chest	5	3	3	2	5	3.60
Left chest	5	3	3	2	5	3.60
Head						
Temporal (axial)—midbrain	4	4	5	4	NA	4.25
Temporal (axial)—falx and ventricles	4	4	5	4	NA	4.25
Frontal (axial)	1	2	2	2	NA	1.75
Coronal	1	2	2	NA	NA	1.67
Sagittal	4	4	4	NA	NA	4.00
Neck						
C-spine (lateral-longitudinal)	3	3	3	3	NA	3.00
C-spine (lateral cross-section)	2	3	3	2	3	2.60
C-spine (AP paratracheal-longitudinal)	2	3	3	2	3	2.60
C-spine (PA longitudinal)	3	3	3	3	3	3.00
C-spine (PA cross-section)	3	3	3	3	3	3.00
Trachea (cross-section)	4	5	5	3	4	4.20
Trachea (longitudinal)	NA	5	5	3	4	4.25
Vascular (carotid/jugular–cross-section)	4	5	5	4	5	4.60
Vascular (carotid/jugular-longitudinal)	5	5	5	4	5	4.80
Chest						
Carina (longitudinal)	5	3	3	3	4	3.60
Carina (cross-section)	NA	5	5	3	NA	4.33
Mediastinum (parasternal-right)	4	2	2	NA	NA	2.67
Mediastinum (parasternal-left)	NA	4	4	NA	5	4.33
Thoracic spine	NA	4	4	2	NA	3.33
Clavical	4	5	5	5	5	4.80
Subclavian vessels	4	5	5	4	5	4.60
Abdomen/pelvis						
Gall bladder	5	5	5	4	5	4.80
Liver	5	5	5	4	5	4.80
Spleen	4	5	5	4	5	4.60
Pancreas	5	2	2	3	4	3.20
Right kidney	5	5	5	4	5	4.80
Left kidney	5	5	5	4	5	4.80
Aorta/vena cava (cross-section)	4	5	5	4	5	4.60
Aorta (longitudinal)	5	5	5	4	5	4.80
Iliac arteries	5	5	5	4	5	4.80
Vena cava (longitudinal)	5	5	5	4	5	4.80
Right renal artery	4	5	5	4	NA	4.50
Left renal artery	4	3	3	4	NA	3.50
Lumbar spine	4	5	5	4	3	4.20
Bladder	5	5	5	5	4	4.80
Prostate (male)	5	5	5	5	4	4.80
Uterus-ovaries (female)	5	4	4	4	NA	4.25
Pubic symphysis	4	4	4	4	4	4.00
Arm						
Humerus (head/neck)	4	NA	NA	3	5	4.00
Midshaft humerus (longitudinal)	5	NA	NA	4	5	4.67
Midshaft humerus (cross-section)	4	NA	NA	3	5	4.00
Lower humerus (cross-section)	5	NA	NA	3	5	4.33
Upper portion of the arm vascular and soft tissue	4	NA	NA	2	5	3.67
Upper radius/ulna (cross-section)	3	NA	NA	3	5	3.67
Radius (longitudinal)	4	NA	NA	4	5	4.33
Ulna (longitudinal)	4	NA	NA	4	5	4.33
Lower radius/ulna (cross-section)	4	NA	NA	4	5	4.33
Carpal tunnel	5	NA	NA	4	5	4.67
Lower portion of the arm vascular and soft tissue	4	NA	NA	4	5	4.33
Leg						
Femur (head and neck)	4	NA	NA	4	5	4.33
Midshaft femur (longitudinal)	4	NA	NA	4	5	4.33
Midshaft femur (cross-section)	4	NA	NA	4	5	4.33
Lower femur (cross-section)	4	NA	NA	4	5	4.33
Upper portion of the leg vascular and soft tissue	5	NA	NA	4	5	4.67
Upper tibia/fibula (cross-section)	4	NA	NA	4	5	4.33
Midshaft tibia (longitudinal)	5	NA	NA	4	5	4.67
Midshaft fibula (longitudinal)	5	NA	NA	4	5	4.67
Lower portion of the leg vascular and soft tissue	5	NA	NA	4	5	4.67

Each reviewer was asked to independently assess the image quality for each of the 65 predefined views of normal anatomy. Imaging settings and probe placements that resulted in average scored ≥3 are considered adequate for inclusion in the CET FAST trauma imaging protocol being developed for the US military.

AVG, average; NA, not applicable; eFAST, Extended Focused Assessment with Sonography for Trauma; AP, Anterior-Posterior; PA, Posterior-Anterior

**Figure 4:**
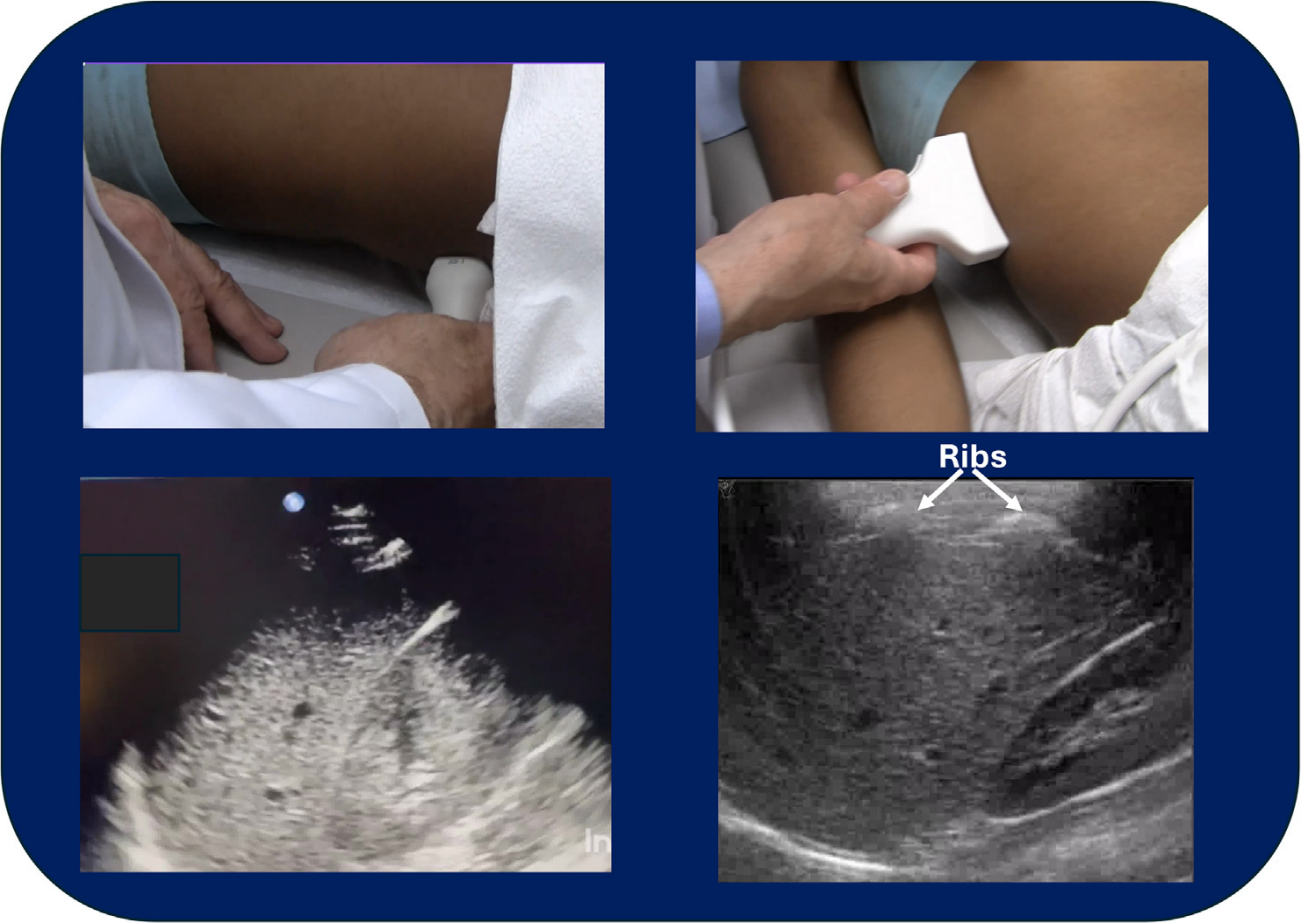
Comparison of images of standard eFAST views of the right upper quadrant. The probe placement photos in the upper half of the figure demonstrate the differences between the probe positions needed to obtain clear views of the right upper quadrant using as standard curved linear ultrasound probe (Phillips IU22 ultrasound system) and the K3900 computer echo tomography system and the concave 3 Mhz MAUI transducer. The lower half of the figure are the images obtained using the 2 systems. Again, with the K3900, the ribs can be easily identified but without the shadowing that would be expected with conventional ultrasound.

Figure 4 is a comparison of standard eFAST images of the right upper quadrant obtained using a standard Phillips IU22 ultrasound and the K3900 CET system demonstrating both the comparability of the images obtained with the CET system.

**Figure 5:**
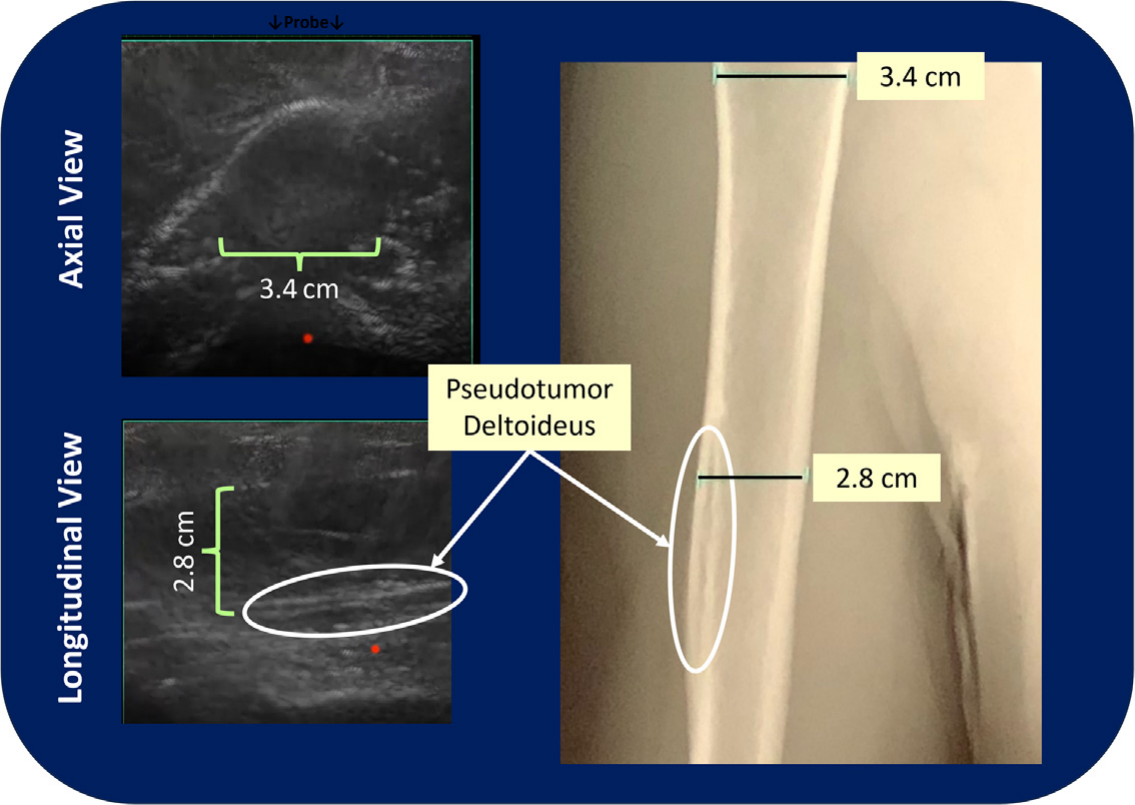
Comparison of long bone images obtained using computer echo tomography (CET) and standard x-ray. To demonstrate the anatomic fidelity of CET, images of the right humerus of an adult male volunteer were obtained using both standard x-ray and the K3900. The measurements obtained were generated by the software of the 2 systems with an experienced radiologist marking the distances to be measured. Serendipitously, the volunteer was found to have a normal anatomic variant known as *pseudotumor deltoideus,* which is seen as a cortical lucency below the insertion of the deltoid muscle on x-ray. Identification of this unusual finding using CET imaging suggests that visualization of nondisplaced fractures of long bones may also be possible.

Figure 5 is a comparison of axial and longitudinal CET views of the humerus of a normal volunteer imaged under the same protocol but outside of the phase 0 baseline study. The images obtained from this volunteer were used to demonstrate the anatomic fidelity of the images obtained using the K3900 as part of MAUIs FDA submission. Serendipitously, the subject was found to have a *pseudotumor deltoideus* on x-ray, which allowed us to assess the ability of CET to identify such lesions. The ability to visualize this cortical defect by CET suggests that nondisplaced long bone fractures may also be visualized.

### 3.1 Limitations

There are 3 significant limitations to this study.

(1) The number of subjects (6) was inadequate to assess the effects of age, sex (biological sex assigned at birth), and body habitus on image acquisition and interpretation.
(2) Only normal healthy volunteers were imaged. No actual pathology was included in the assessment of image quality. As a result, we have no ability to comment upon the sensitivity or specificity of making specific diagnoses.
(3) Visualization and measurement of vascular flow (equivalent to conventional Doppler capability) was not available when this study was conducted. This feature is currently being incorporated into the MAUI system.

## 4 Discussion

CET-based imaging was developed primarily to address the limitations of conventional ultrasound with respect to the ability to image through and around barriers such as bone, gas, or foreign bodies. The images obtained using the K3900 suggest that CET may overcome the problems caused by these barriers and provide for a significant improvement in the assessment of traumatic injuries at the bedside.

When comparing the adequacy of image acquisition for the standard views of the eFAST examination, CET imaging of the abdomen/pelvis appeared to be comparable to traditional ultrasound with respect to image quality (American College of Emergency Physicians [ACEP] image grades ≥4.0, Fig 4), but image acquisition may be significantly easier for less experienced practitioners because of the ability to image through the ribs without having to find optimal intercostal or subcostal windows. Of note, standard FAST views of the heart were considered suboptimal (subxiphoid view ACEP image grade = 2.25) due to the probe’s size and concave design making it difficult to push the probe under the rib cage and obtain adequate probe-skin contact. However, by replacing the subxiphoid view with a left parasternal view, through the ribs, evaluation of the heart and pericardium became much more acceptable (Fig 3, right; ACEP image grade = 4.6).

Results obtained when imaging the cranial vault were mixed with respect to image quality. Temporal axial imaging through the squamosal bone yielded the best results with visualization of the ipsilateral and contralateral cerebral hemispheres and subdural spaces, as well as the midbrain and falx (Fig 2). These images suggest that when fully developed, CET imaging may be able to image intracranial pathologies (bleeds, foreign bodies, tumors, midline shifts, etc) in the absence of CT and/or MRI and, potentially, provide for real-time guidance of external ventricular drain (EVD) placement.

Other areas where CET imaging may prove to be superior to traditional ultrasound is imaging of the mediastinum, where the ability to assess endotracheal tube placement with visualization of the trachea, carina, and right and left mainstem bronchi below the sternum being possible (Fig 3, left). And, although fracture diagnosis with conventional ultrasound is possible,^8^^,^^9^ improved visualization of the long bones of the arm (Fig 5) and leg and the spine may allow for both the diagnosis and management (image-directed reduction and external fixation) of fractures in resource-limited environments, a major advantage for role 1 and role 2 combat casualty care, to become a practical reality.

The current study (phase 0) was the first in a 3-phase project with phases 1 and 2 being conducted at the University of Maryland Shock Trauma Center. Phase 1, which is currently in progress, is intended to provide information on the effects of different body types on image acquisition and quality. At least 25 different individuals for each of the anatomic regions will be evaluated to better understand how age, sex, and body habitus impacts CET imaging.

Phase 2 is designed to assess the ability of CET imaging to visualize known pathology (subdural hematomas, pneumothorax, hemopericardium, intra-abdominal bleeds, displaced and nondisplaced long bone fractures, etc) identified using standard-of-care imaging techniques (CT and x-ray) as well as for providing real-time guidance and/or placement verification for device placement (endotracheal tubes, central lines, chest tubes, REBOA resuscitative endovascular balloon occlusion of the aorta (REBOA) catheters, and EVDs) in the acute setting.

CET represents a categoric change in ultrasound imaging that offers the potential for significantly improving patient management in emergent settings, regardless of the availability of other imaging modalities. The triage of patients due to mass casualty events may also benefit from the availability of CET imaging. These initial findings are encouraging with respect to the potential for developing a true “head-to-toe” CET FAST examination protocol capable of providing rapid and accurate diagnosis, triage, and management of trauma victims in rural and/or other resource-limited, and/or austere environments. Continued evaluation of CET imaging for the assessment and management of trauma is warranted.

## Author Contributions

JC (the overall project PI), designed the study, wrote the majority of the manuscript, and obtained research funding from the US Army Medical Research and Development Command, Combat Casualty Care Research Program. MC and DN were the sonographers who conducted the imaging. MB, PKC, RK, JM, and MM were the independent expert clinicians that scored the images obtained using the K3900. EP is an emergency physician and sonography expert at University of Maryland Baltimore County, a named investigator on the overall project, and the primary editor of the manuscript. DS was the founder and CEO of MAUI Imaging and is the co-inventor of Computed Echo Tomography. He was primarily responsible for drafting the manuscript sections describing CET.

## Funding and Support

Supported by the Office of the Assistant Secretary of Defense for Health Affairs through the Combat Casualty Care Research Program under Award No. HT9425-23-3-0002. The views and conclusions contained herein are those of the authors and should not be interpreted as necessarily representing the official policies or endorsements, either expressed or implied, of the US Government.

## Conflict of Interest

JC, MC, DN received financial support and travel reimbursement from US Army Medical Research and Development Command (USAMRDC) and have served as consultants or advisors and hold equity or stocks in MAUI Imaging, Inc. RK and EP received financial support from USAMRDC. JM holds equity or stocks in MAUI Imaging, Inc. DS received financial support and travel reimbursement from USAMRDC, has been employed by and holds equity or stocks in MAUI Imaging, Inc, and is a named inventor on multiple patents issued to MAUI Imaging, Inc. The other authors have no conflicts of interest to declare.
